# Sulfur Starvation in Extremophilic Microalga *Galdieria sulphuraria*: Can Glutathione Contribute to Stress Tolerance?

**DOI:** 10.3390/plants11040481

**Published:** 2022-02-10

**Authors:** Giovanna Salbitani, Angela Perrone, Luigi Rosati, Carmen Laezza, Simona Carfagna

**Affiliations:** 1 Dipartimento di Biologia, Università di Napoli Federico II, Via Cinthia 21, 80126 Naples, Italy; giovanna.salbitani@unina.it (G.S.); ange.perrone@studenti.unina.it (A.P.); luigi.rosati@unina.it (L.R.); 2 Dipartimento di Agraria, Università di Napoli Federico II, Portici, 80055 Naples, Italy; carmen.laezza@unina.it

**Keywords:** *Cyanidiophyceae*, *Galdieria sulphuraria*, glutathione, superoxide dismutase (SOD), sulfur starvation

## Abstract

This study reports the effects of sulfur (S) deprivation in cultures of *Galdieria sulphuraria* (Cyanidiophyceae). *Galdieria* is a unicellular red alga that usually grows, forming biomats on rocks, in S-rich environments. These are volcanic areas, where S is widespread since H_2_S is the prevalent form of gas. The glutathione content in *Galdieria sulphuraria* is much higher than that found in the green algae and even under conditions of S deprivation for 7 days, it remains high. On the other hand, the S deprivation causes a decrease in the total protein content and a significant increase in soluble protein fraction. This suggests that in the conditions of S starvation, the synthesis of enzymatic proteins, that metabolically support the cell in the condition of nutritional stress, could be up regulated. Among these enzymatic proteins, those involved in cell detoxification, due to the accumulation of ROS species, have been counted.

## 1. Introduction

There is no doubt that in recent years, microalgae have attracted the attention of researchers for their multiplicity of uses in different fields. Microalgae belonging to the genus *Galdieria* have aroused greater interest since they synthesize molecules (e.g., C-phycocyanin or glycogen) by interesting application in medicine, biotechnology, pharmacology and nutraceutical [1,2,3]. Among the many species of microalgae that have aroused greater interest, the genus *Galdieria*, is included. *Galdieria* sp. are polyextremophiles organisms surviving in acidic geothermal environments, found throughout the world and sharing similar characteristics [4]. In particular, *Galdieria sulphuraria* grows with acid pH (between 0.5 and 4.0) and at temperatures of up to 56 °C, i.e., close to the upper limit for eukaryotic life [5,6]. *G. sulphuraria* thrives in volcanic hot sulfur springs where the hydrogen sulphide (H_2_S) is progressively oxidised to sulfur and H_2_SO_4_. Therefore, *G. sulphuraria* may be considered a “sulfurous” microalga since it is also highly dependent on S for its mineral nutrition. While several soils around the world have had limited S content [7,8], some terrestrial environments are rich in S and therefore the organisms that populate them survive in the presence of excessive amounts of inorganic sulfur. These environments are typically volcanic ones and calderas, where H_2_S is the prevalent form of gaseous S in the fumarolic fluids and S is widespread in the soils [9].

Sulfur is an essential macronutrient for all plant cells. It is majorly absorbed as sulphate from the environment and translocated to plastids where it is assimilated into S organic metabolites. Therefore, plants are also capable of utilizing other S sources, such as various atmospheric S containing gases [10].

S assimilation in plant cell can be summarized in four steps: (1) sulfate uptake; (2) sulfate activation; (3) reduction of activated sulfate; (4) synthesis of S-containing molecules. The last step of S assimilation stages the cysteine (Cys) biosynthesis through O-acetylserine(thiol)lyase (OASTL, EC 4.2.99.8) enzymes [11]. From Cys derive many other essential S metabolites, including amino acids (methionine), vitamins, iron-sulfur clusters, phytochelatins and glutathione. The thiol groups (-SH) of the Cys residues in proteins form disulfide bridges, important for the protein structural maintenance. They are also important for enzyme activities and their regulations. Thiol residues in glutathione (GSH) are essential for detoxification of Reactive Oxygen Species (ROS) occurring in plant cells in a plethora of abiotic stress conditions [12,13,14,15].

Sulfur is of great importance for the plant metabolism so that, under its limited availability, various metabolic arrangements take place: from its uptake, mediated by specific transporters [16,17], up to its assimilation into the amino acid Cys [12]. Therefore, an environment that is too rich in sulfur represents a stressful, albeit rare, condition for plants. H_2_S is a phytotoxic molecule causing in plants accumulation of ROS and oxidative stress [18]. A natural S-rich habitat is a unique feature of oxygenic photosynthesizing organisms and thus merits consideration.

In the last few years, sulfur uptake, its assimilation and the effects of its deprivation have been widely and extensively treated in plants and in microalgae. Several studies on microalgae have revealed that S transporters and some enzymes of the S assimilation pathway show a negative feedback mechanism driven by S demand [19]. That is, for instance, in *Chlorella sorokiniana* the cysteine synthesis is repressed when sulfate is available and it is activated by S starvation [14,20]. Also, in *Chlamydomonas reinhardtii* the sulfate transport activity increased in S limited cells [21,22]. Our recent research in the red microalgae *Galdieria phlegrea* [23] demonstrated that S deprivation for 24 h affects cellular metabolism depending on autotrophic or heterotrophic growth conditions.

In this study, the relationship between S deprivation and cell metabolism was investigated in the microalgae *Galdieria sulphuraria* for a period of 7 days. The idea of this research is to evaluate the metabolic changes occurring in this microorganism, adapted to grow in an environment rich in S, under prolonged deficiency of sulfur. The effects of S deficiency on cellular growth and on protein contents were considered. To assess changes of cellular redox state in response to S starvation, the levels of oxidized glutathione, hydrogen peroxide, and activity of the SOD enzymes were investigated.

## 2. Materials and Methods

### 2.1. Algal Strain and Growth Media

Experiments were performed with axenic cultures of *Galdieria sulphuraria* (strain 074G) from the ACUF collection of the Department of Biology of the University of Federico II, Naples, Italy (http://www.acuf.net/index.php?lang=en, accessed on 5 January 2022). *Galdieria* control cells were grown in Allen’s medium [24] containing 10 mmol L^−1^ (NH_4_)_2_SO_4_ as a nitrogen source. The initial algal concentration was around 5 × 10^6^ cells mL^−1^. The flasks were placed on a Lab shaker under continuous irradiance (150 μE m^−2^ s^−1^) provided by daylight LED lamps. Carbon dioxide was supplemented by sparging filtered air into the medium. The pH was set at 1.5 by sulfuric acid whereas the temperature was maintained at 35 ± 1 °C.

To prepare S starved cultures, cells grown in a complete Allen’s medium, were harvested during the logarithmic growth phase (0.8 ˂ OD_800_ ˂ 1.0) by low-speed centrifugation at 4000× *g* for 10 min and then washed twice with S-free Allen’s medium. Then, the supernatant was removed and the algal pellets were re-suspended in S-free Allen’s medium. In the S-free Allen’s medium (NH_4_)_3_PO_4_, MgCl_2_ and FeCl replaced their respective sulfuric acid salts ((NH_4_)_2_SO_4_, MgSO_4_, FeSO_4_) without changing the molarity of the other individual ions. The pH 1.5 was adjusted adding hydrochloric acid (pH) instead of sulfuric acid.

The cultures were sampled daily, and growth was followed by measuring the optical density of the cultures at 800 nm with a spectrophotometer (Thermo, Helios Biomate 5, UK). The cell number and cell size were determined by a Countess II FL Automated Cell Counter (Thermo Fisher Scientific Inc., Waltham, MA, USA).

### 2.2. Imaging

All microscopic observations were conducted in triplicate on 1 mL cellular culture both for the control and the S deprived. Fresh samples were deposited on a slide and immediately observed at microscope. For light and fluorescence (red autofluorescence) microscopy analysis, the images were captured with a camera attached to an IBM computer running the Kontron Elektronik KS 300 image analysis system (Carl Zeiss MicroImaging s.p.a., Milan, Italy).

### 2.3. Protein Extraction and Determination

Algal cells (300 mL of culture) were harvested by low-speed centrifugation (4000× *g* for 5 min). The packed cells were re-suspended in 2 mL of cold extraction buffer (50 mM Tris-HCl, pH 8.00; 1 mM Na-EDTA; 2 mM DTT) and were lysed by passaging at 1000 psi through French Pressure Cell (Aminco, Woonsocket, RI, USA) and centrifuged at 12,000 rpm for 30 min at 4 °C; the clear supernatant was used as crude extract. Total proteins were determined spectrophotometrically using the Bradford-Solution (PanReac, AppliChem, A6932) based on the Bradford method [25] with bovine serum albumin as the standard. The proteins were expressed as mg cell^−1^ (10^−6^).

For the determination of water-soluble proteins, aliquots (100 mL) of algal suspension were subjected to centrifuging (4000× *g* for 5 min). The algal pellet was re-suspended in 5 mL distilled water and then boiled for 2 h. After refrigeration, the water extract was centrifuged at 15,000× *g* at 4 °C for 15 min. The clear supernatant was used for the spectrophotometric determination of soluble protein concentration as previously described [26].

### 2.4. Ammonium Analysis in Growth Medium

For the estimation of ammonium content in the algal growth medium, supernatants were collected after low-speed centrifugation (4000× *g* for 4 min) of algal culture (1 mL). The residual ammonium in the supernatant was estimated based on a Nessler method modified for microalgae as previously described in detail [2].

### 2.5. H_2_O_2_ Extraction and Determination

An aliquot of 100 mL of algal culture was harvested using centrifugation (4000× *g* for 10 min). The pellet was re-suspended in 2 mL of 0.1% TCA (Trichloroacetic-acid) and then lysed by passaging at 1000 psi through a French pressure cell. The homogenate was centrifuged at 12,000× *g* for 30 min at 4 °C and the clear supernatant (crude extract) was used for H_2_O_2_ determination. Crude extract (250 µL) was added to 250 µL of 10 mM phosphate-buffer pH 7.00 and 500 µL of 1 M KI. The reaction mixtures were measured at 390 nm. The H_2_O_2_ concentration was calculated using a linear calibration curve with H_2_O_2_ solutions.

### 2.6. Superoxide Dismutase (SOD) Activity

An aliquot (10–50 µL) of crude extract (see above) was added to the SOD reaction mixture (final volume of 1 mL) containing 50 mM phosphate buffer (pH 7.7), 0.1 mM EDTA, 13 mM methionine, 75 μM nitroblue tetrazolium (NBT), and 2 μM riboflavin for the SOD assay. The reaction mixture was exposed for 15 min at a light intensity of 350 μmol m^−2^ s^−1^ and then the absorbance was monitored at 550 nm. One unit of SOD activity was defined as the amount of enzyme required to cause 50% inhibition of NBT reduction.

### 2.7. Glutathione Extraction and Determination

Cellular pellet from 200 mL of algal culture was re-suspended in 3 mL of 5% sulfosalicylic acid. Cells were lysed by a passage at 1000 psi through French Pressure Cell (Aminco, Woonsocket, RI, USA) and centrifuged at 16,000 rpm for 20 min at 4 °C; the clear supernatant was used as crude extract. The concentration of GSH was determined as previously described [1] and expressed as nmol cell^−1^ (10^−6^).

### 2.8. Statistical Analysis

Experimental data analyses were made using Sigmaplot 14 software. Data of mean ± SE of three independent experiments were presented. The statistical analysis was performed by one-way ANOVA with a Tukey post-hoc test to determine differences between control and S starved cells.

## 3. Results

### 3.1. Growth and Microscopy Observation

**Figure 1:**
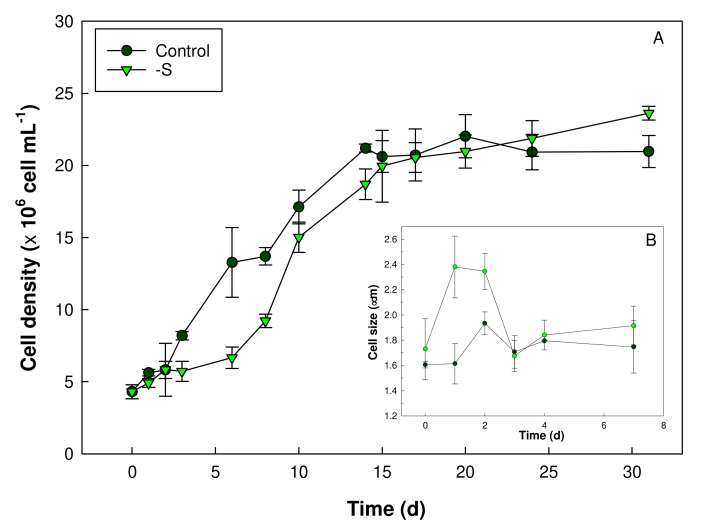
Cell density (**A**) and average cell diameter (**B**) of *Galdieria sulphuraria* grown in a complete (control) and a S deprived (-S) medium. The cell number and size were determined by a Countess II FL Automated Cell Counter (Thermo Fisher Scientific). The number of cells was assessed for a period of 30 days. The cell size was measured for 7 days. Error bars represent SD (*n* = 4).

Cultures of *Galdieria sulphuraria* were maintained for a period of 30 days in an S deficient medium or in Allen’s medium containing all nutrients (control) (Figure 1A). The number of cells per mL of culture was not significantly different in both cultures starting approximately from 10 days. During the 10 days, the cell growth of the S deprived cultures was slower than the controlled one.

The difference in the average cell size is shown in Figure 1B. In S starved cells, in the first two days from the start of the S starvation, an increase in average cell diameter was measured in respect to control (Figure 1B). For the rest of the experiment, the cell size in both conditions was comparable with each other.

**Figure 2:**
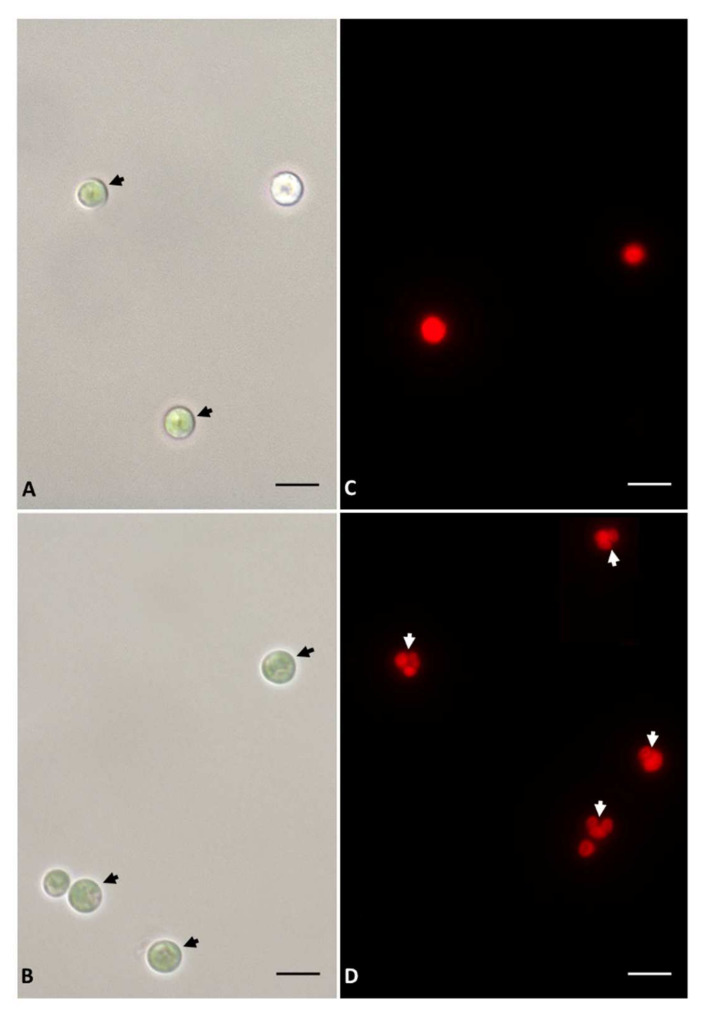
Images in light and in fluorescence microscopy of *Galdieria sulphuraria* cells collected after 2 days from the start of experiment. Light microscopy: (**A**): control cells. (**B**): S starved cells. In both analysed samples are evident cells with a rounded shape (dark arrow). Fluorescence microscopy: (**C**): control cells. (**D**): S starved cells. In S starved cells some daughter cells (autospores) before hatching (white arrow) are apparent. (**A**–**D**): Bars correspond to 5 µm.

Light and fluorescence microscopy observations were performed on cells of *Galdieria sulphuraria* collected in the two different culture conditions and at two different times (2 and 7 days). The samples collected after 2 days, both control and S starved (Figure 2A,B), showed in light microscopy round cells without difference between the two conditions. By fluorescence microscopy observations, due to the autofluorescence of chlorophylls contained into the chloroplasts, we noted several S starved cells not fully divide. Furthermore, some daughter cells (autospores) before hatching (Figure 2D) were not visible in control samples (Figure 2C).

**Figure 3:**
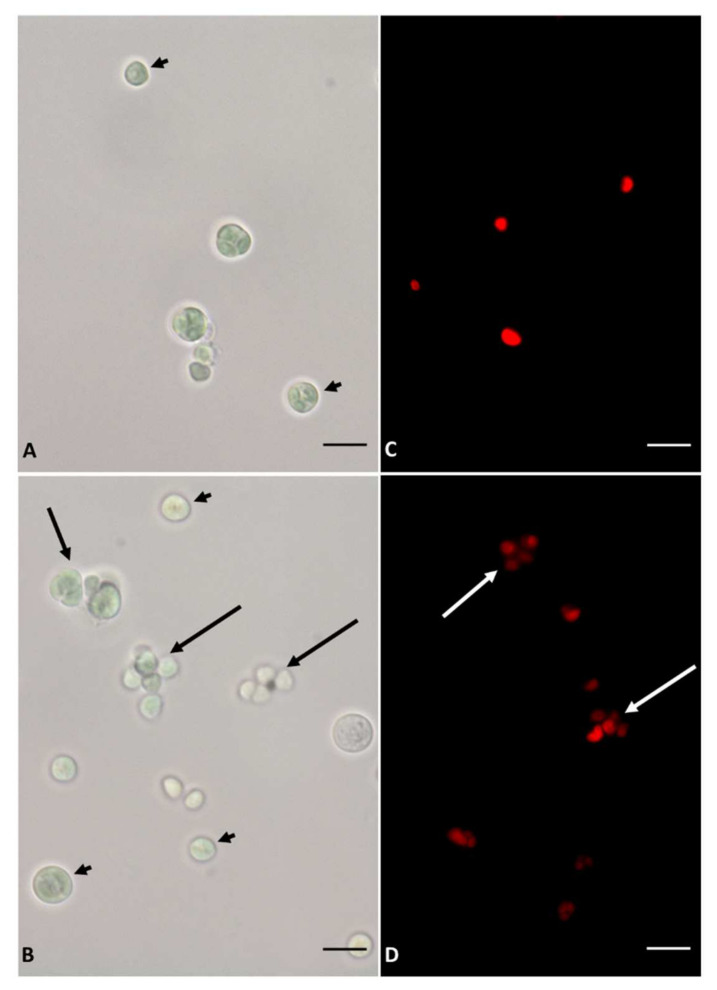
Images in light and in fluorescence microscopy of *Galdieria sulphuraria* cells collected after 7 days from the start of the experiment. Light microscopy: (**A**): control cells. (**B**): S starved cells. In both analysed samples, cells with a rounded shape (black arrow) are evident. In the S starved (**B**) cellular aggregates (black long arrow) are evident. Fluorescence microscopy: (**C**): control cells. (**D**): S starved cells. In S starved cells cellular aggregates (white long arrow) are evident. (**A**–**D**): Bars correspond to 5 µm.

After 7 days the cells showed a similar morphological square (Figure 3), however, in the S starved cells, both light and fluorescence microscopy, a greater number of cellular aggregates was highlighted.

### 3.2. Protein Contents of the Cells

**Figure 4:**
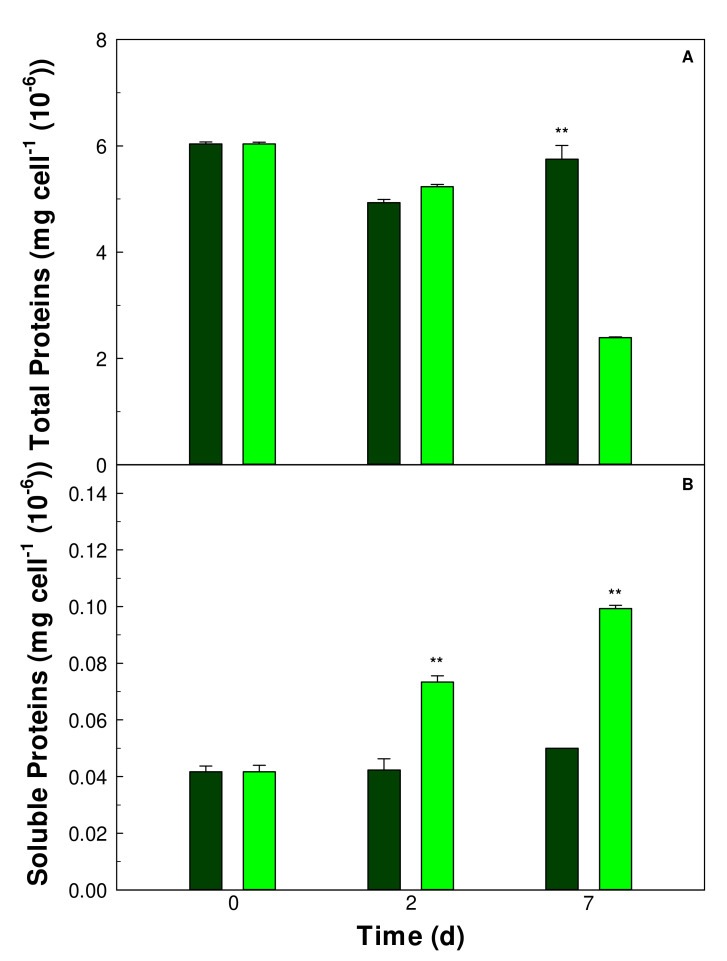
Total (**A**) and soluble (**B**) protein contents in *Galdieria sulphuraria* control (dark green) and S starved (-S, light green) cells. The proteins were measured at 0, 2 and 7 days from the start of the experiment. Error bars represent SD (*n* = 3). Significant differences between control and -S were determined by Tukey HSD test and indicated by asterisks (** *p* ˂ 0.01).

In cells of *Galdieria sulphuraria* maintained in an S deficient medium or in Allen’s medium containing all nutrients (control), the total and soluble protein contents were evaluated. In the first 7 days of cultivation, the total protein content was affected by the S deprivation in *Galdieria sulphuraria* cells (Figure 4A). At 7 days, in the S starved cells total proteins become 2.39 ± 0.017 mg cell^−1^ (10^−6^), reducing to one third from the initial value (6.0 ± 0.03 mg cell^−1^ (10^−6^)).

However, despite the reduction in total protein, the soluble proteins fraction increased over 7 days of cultivation in S starved cells (0.99 ± 0.001 mg cell^−1^ (10^−6^)) compared to control cells (0.005 ± 0.0001 mg cell^−1^ (10^−6^)) (Figure 4B).

### 3.3. Ammonium Uptake

**Figure 5:**
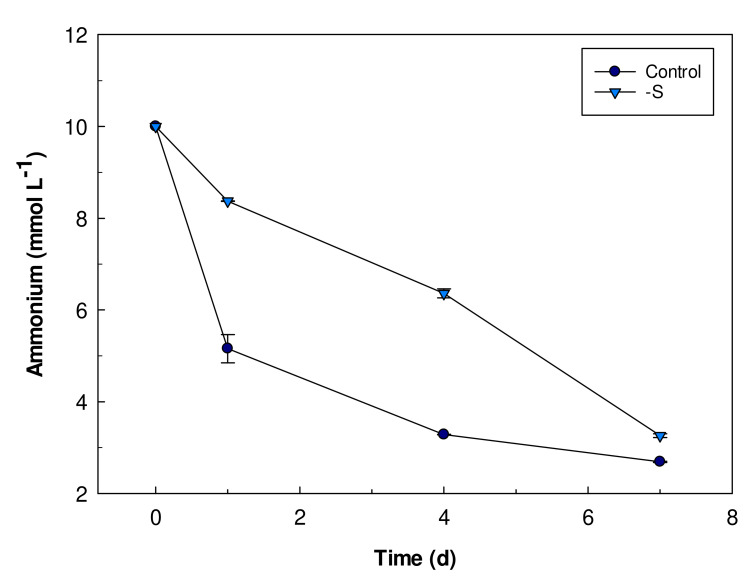
Ammonium content (mmol L^−1^) in culture media of control and S starved (-S) cells. The residual ammonium in the cultures was estimated based on a Nessler method. Error bars represent SD (*n* = 3).

The absorption of the nitrogen present in the culture medium in the form of ammonium was monitored during the experiment. Control and S starved cells of *Galdieria sulphuraria* absorb ammonium from their medium with a different pattern and rate (Figure 5). Control cells absorbed half of the ammonium available in the medium in the first day of cultivation while S starved cells took up only 20% at the same time. After 7 days, in both cultures, the cells absorbed 70% of the ammonium present in the growth medium.

### 3.4. Hydrogen Peroxide Content

**Figure 6:**
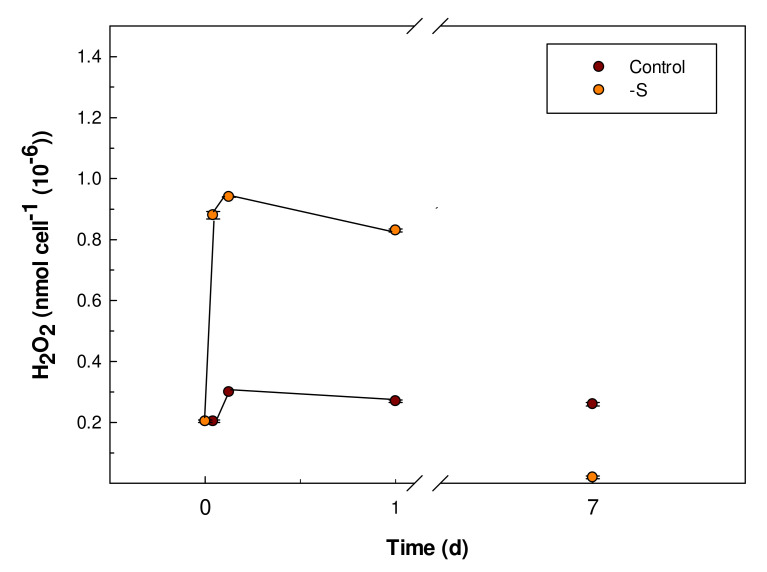
Hydrogen peroxide intracellular contents in control and S starved (-S) cells of *Galdieria sulphuraria*. The H_2_O_2_ levels were measured during the experiment at 0, 1, 3 h and at 1 and 7 days and expressed as nmol cell^−1^ × 10^6^. Error bars represent SD (*n* = 3).

To evaluate the impact of S starvation in *G. sulphuraria* on the occurrence of oxidative stress, the intracellular content of hydrogen peroxide was determined, like time course, during the S starvation for a maximum period of 7 days. The H_2_O_2_ levels significantly increased after 1 h from the beginning of the S starvation, passing from 0.2 ± 0.004 nmol cell^−1^ (10^−6^) to 0.9 ± 0.001 nmol cell^−1^ (10^−6^). The same trend of increase was maintained up to the three hours of S starvation but at the end of the experiment (7 days), the H_2_O_2_ content decreased to a value lower than that of control cells (Figure 6).

### 3.5. Thiol Contents of the Cells

**Figure 7:**
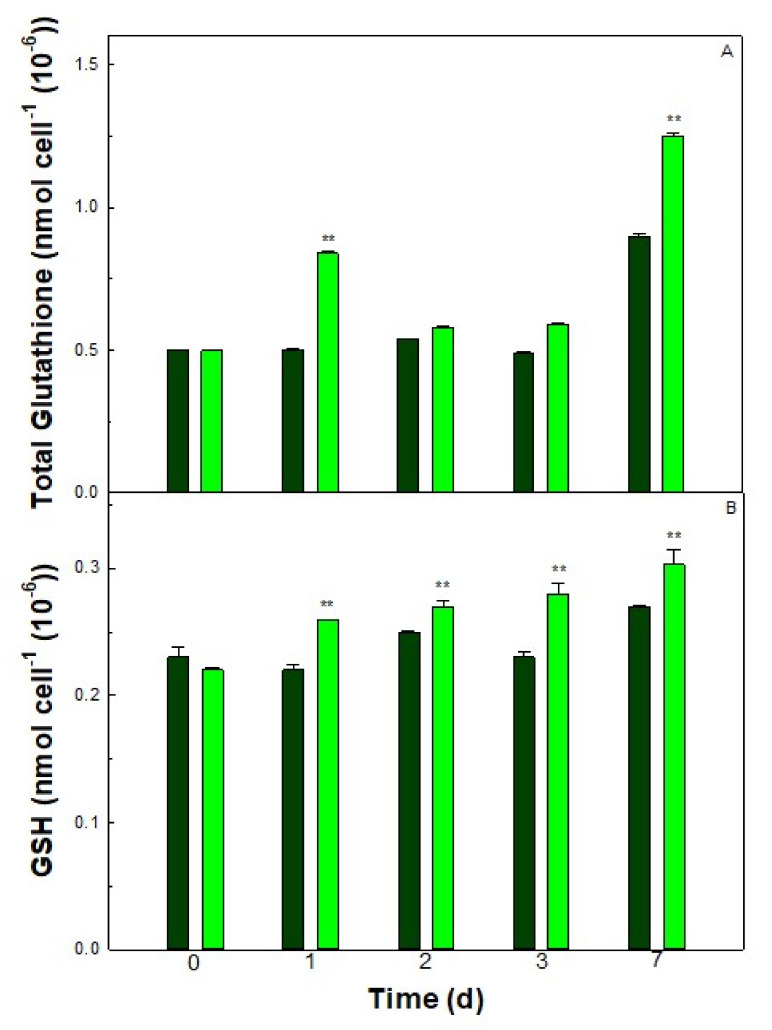
Total (**A**) and reduced (**B**) glutathione in control (dark green) and S starved (-S, light green) cells of *Galdieria sulphuraria*. Error bars represent SD (*n* = 3). Significant differences between control and -S were determined by Tukey HSD test and indicated by asterisks (** *p* ˂ 0.01).

In cells of *Galdieria sulphuraria* grown in an S deficient medium or in Allen’s medium containing all nutrients (control) the total and reduced glutathione contents were evaluated. The total glutathione was 0.50 ± 0.001 nmol cell^−1^ (10^−6^) in control cells. Therefore, in cells cultured under conditions of S starvation, the total glutathione contents resulted in a higher number with respect to control cells after one day from the start of S starvation (0.84 ± 0.005 nmol cell^−1^ (10^−6^)). After 7 days, it resulted in 1.25 ± 0.008 nmol cell^−1^ (10^−6^). The reduced glutathione (GSH) significantly increased in S starved cells, reaching the concentration of 0.3 ± 0.01 (Figure 7), at 7 days.

### 3.6. The Superoxide Dismutase (SOD) Enzyme Activity

**Figure 8:**
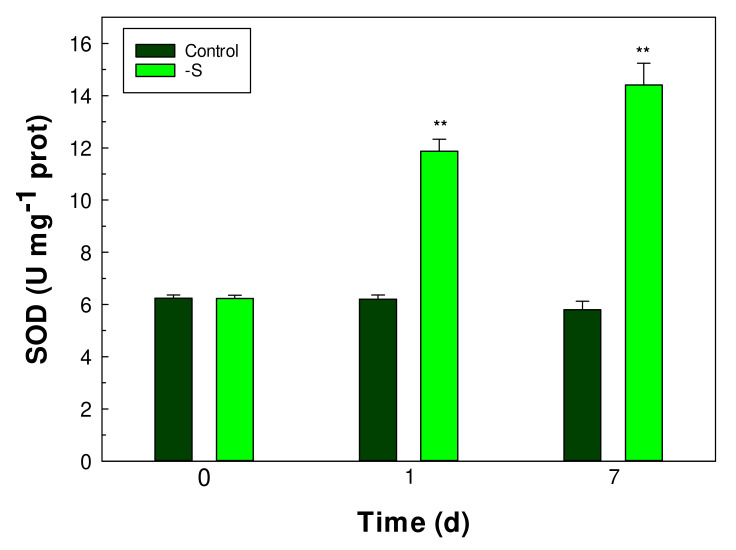
SOD activity in control and S starved (-S) cells of *Galdieria sulphuraria*. Error bars represent SD (*n* = 3). Significant differences between control and -S were determined by Tukey HSD test and indicated by asterisks (** *p* ˂ 0.01).

The superoxide dismutase (SOD) constitutes an important antioxidant defense against oxidative stress. It catalyzes the dismutation of superoxide (O_2_^−^) to hydrogen peroxide (H_2_O_2_) and O_2_. The activity of SOD was found to be 6.2 ± 0.1 U mg prot^−1^ in control cells of *Galdieria sulphuraria*. However, the SOD activity increased in a time-dependent manner in the cells under S starvation and remained unchanged in the control cells. After 1 day from the start of S starvation, the SOD activity was 11.9 ± 0.5 U mg prot^−1^, double that of control cells. Again, after 7 days, the SOD activity increased to 14.4 ± 0.83 U mg prot^−1^ in S starved cells, a value far greater than in the control cells (5.8 ± 0.32 U mg prot^−1^) (Figure 8).

## 4. Discussion

The growth of plants and algae is strongly influenced by the availabilty of nutrients. Essential macronutrients such as sulfur, nitrogen or phosphorus affects microalgal growth; a shortage of these nutrients results in the slowdown/arrest of cell division [27,28]. In *Galdieria sulphuraria* cultures, the lack of S in the medium, alters cellular growth in the first 7 days of deprivation. In fact, the cellular growth rate resulted lower in the S deficiency than in the control condition. After 7 days, the S starved cells growth returns to a rate comparable to that of control cultures. Hence, S deficiency impairs cell growth in the first few days preceding a period of adaptation and metabolic response of the cells. Interestingly, in the first two days of starvation, *G. sulphuraria* cells exhibited a larger diameter in respect of the control ones. This increase in cell volume is an aspect repeatedly reported in literature referring to microalgae subjected to nutritional deficiency and attributed to the accumulation of reserve substances such as sugars or lipids [29,30]. However, all algae during their cell cycle, undergo a period of cellular enlargement, even more than 10-fold under favorable conditions, that comes before the formation of autospores and then of daughter cells. Moreover, unfavorable growth conditions might force the cells to undergo repeated mitotic division without subsequent cytokinesis, thus resulting in increased cell size [31]. Our fluorescence microscopy observations revealed several cells of *Galdieria* S starved, not yet fully divided, which could explain the apparent increase in cell diameter. Instead, after protracted S starvation, that was 7 days long, the mean cell diameter was not significantly different between S starved and control cells but starved cells appeared aggregated. Several articles report this effect in microalgae induced by stressful conditions [32]. Under sulfur deprivation in the green alga, *Chlamydomonas reinhardtii*, the most prominent model organism, changes with the profile of extracellular proteins, modifying the charge of the cell surface which could induce a cellular aggregation [33]. We can hypothesize a phenomenon of cellular aggregation also in *Galdieria sulphuraria*, but more in-depth microscopy analyzis will certainly be useful.

In *G. sulphuraria* about 50% of total proteins are insoluble and localized in the cell wall [34]. In this study the protein contents in *Galdieria sulphuraria* cells, considering total proteins and soluble fractions, under S starvation was reported. Total proteins decreased gradually by increasing days of S deprivation culturing. Clearly, in the condition of S starvation, the synthesis of the sulfur amino acids like cysteine and methionine was compromised, influencing in a decisive way the protein synthesis. Proteins in plant cell represent an important sink for reduced sulfur in the form of cysteine [35]. The slowdown of protein synthesis inevitably affects the ability of cells to use inorganic nitrogen, an essential macronutrient for all plant organisms [14]. Both the absorption and assimilation of inorganic nitrogen, in the form of ammonium or nitrate ions, is strictly dependent on the availability of sulfur. In fact, in the S starved cells of *Galdieria sulphuraria* the absorption of ammonium from the culture medium was slowed down compared to control cells. However, our results also showed a significant increase in soluble protein fraction in S starved cells. These results suggest that in the condition of S starvation, the synthesis of enzymatic proteins, that metabolically support the cell in the condition of nutritional stress, could be up regulated. Among these enzymatic proteins, are those involved in cell detoxification, due to the accumulation of ROS species, which have been quantified. The early and rapid increase in the intracellular content of H_2_O_2_ found in the condition of S starvation in *Galdieria sulphuraria* cells could represent an intracellular redox imbalance but also a signaling that leads to the activation of the antioxidant defence system [13,36,37]. A significant increase in SOD activity was observed in *Galdieria sulphuraria* cells one day after the beginning of the S starvation. The rapid H_2_O_2_ increase following the start of S starvation could also be related to the increased activity of SOD.

However, after 7 days of S starvation, the intracellular H_2_O_2_ was at a lower value than that of the control cells.

This reduction can be explained as follows: (1) cellular respiration and photosynthesis, i.e., the main physiological processes involved in the natural production of H_2_O_2_, could decrease in S starved cells [38,39]; (2) the H_2_O_2_ may have been excreted into the culture medium [40], one of the mechanisms of algae to avoid the harmful intracellular accumulation of H_2_O_2_; (3) the H_2_O_2_ content could be related to the activity increases of antioxidant enzymes [13].

In algae and plants, glutathione represents an essential S-containing compound comprising cysteine, the first S-amino acid obtained by sulfur assimilation. In *Galdieria sulphuraria* like in *Galdieria phlegrea* [23], the glutathione content is much higher than that found in the green algae *Chlorella sorokiniana* [13]. This data could suggest that in these “sulfurous” algae of the genus *Galdieria*, there may be more abundant reserves of organic sulfur than in those of other microalgae, in the form of glutathione or even of a protein nature. Being H_2_S a molecule having a phytotoxic effect [20], the efficient assimilation of these gases is important since it could cause the accumulation of ROS and oxidative stress. The increased synthesis of glutathione and antioxidant enzymes activity could protect the cell against oxidative stress.

After the first day of S starvation, the total glutathione level of *G. sulphuraria* increased while a decrease in both Cys and glutathione intracellular levels was previously observed in *C. sorokiniana* cells grown under the same deprived condition [13]. In this respect, it is noteworthy that under S deficiency, in *G. sulphuraria* the glutathione pool increased both in reduced and in oxidized form. In the following days, total glutathione decreased while its reduced form unchanged: this would mean that oxidized glutathione decreased too. Almost certainly, glutathione has effectively buffered the change in the redox state of the cell which occurs immediately upon the beginning of S starvation, as verified by the temporary increase in hydrogen peroxide. After 7 days, total glutathione increased in both control and S starved cells, but total protein content decreased in S starved cells. Under S starvation, we can suppose that: 1. most likely cysteine is directed for the synthesis of glutathione rather than for the synthesis of methionine and proteins; 2. the cysteine required to maintain high levels of glutathione derives from other reserves, such as proteins. Both hypotheses should certainly be verified. It has also been proposed that glutathione can also act as a signal to control sulfate uptake in higher plants [41,42] and Chlorophyceae [43], however it has not yet been studied in Cyanidiophyceae. It has been demonstrated that H_2_S represents an alternative source of sulfur for plants, since contrary to sufate [35,44], it can be fixed and stored in the form of cysteine, glutathione and thiosulfate. It is fair to suggest a similar metabolic route in any photosynthetic organisms and particularly in microalgae inhabiting sulfur springs with high H_2_S emissions. To date, nothing is known about thiosulfate in *Galdieria* or about organic compounds containing sulfur that can act as sulfur reserves into the cell. However, our results suggest that glutathione may play a key role in *Galdieria* to tolerate sulfur deficiency even for several days. Further research will be indispensable to understand the adaptive mechanisms of these photosynthetic organisms in order to survive in environments hostile to other forms of life.

## 5. Conclusions

*Galdieria sulphuraria* represents a very interesting microorganism for understanding the mechanisms of tolerance to stress from deficiency and from excess of sulfur, since both conditions represent a stressful condition for all plant organisms. High levels of glutathione found in *Galdieria* cells could add to metabolic strategies operating in extremophilic organisms.
